# Supporting Clinical Decisions with Rapid Molecular Diagnostic Pneumonia Panel in Pediatric Intensive Care Unit: Single Center Experience in Turkiye

**DOI:** 10.3390/microorganisms11102391

**Published:** 2023-09-25

**Authors:** Gurkan Bozan, Yalcin Kara, Eylem Kiral, Mahmut Can Kizil, Ebru Kacmaz, Tercan Us, Gul Durmaz, Omer Kilic, Ener Cagri Dinleyici

**Affiliations:** 1 Pediatric Intensive Care Unit, Faculty of Medicine, Eskisehir Osmangazi University, Eskisehir 26040, Türkiye; dr_eylem@hotmail.com (E.K.); elvinduru3446@gmail.com (E.K.); timboothtr@yahoo.com (E.C.D.); 2 Pediatric Infectious Disease Unit, Faculty of Medicine, Eskisehir Osmangazi University, Eskisehir 26040, Türkiyemcankizil@hotmail.com (M.C.K.); omerkilic7@yahoo.com (O.K.); 3 Department of Microbiology, Faculty of Medicine, Eskisehir Osmangazi University, Eskisehir 26040, Türkiye

**Keywords:** pneumonia, ventilator-associated pneumonia, rapid molecular diagnostic panel, pediatric intensive care unit, antibiotic resistance

## Abstract

Introduction: Lower respiratory tract infections are the leading cause of morbidity and mortality in children worldwide. It is crucial to promptly conduct diagnostic investigations in order to determine the microbiological cause of pneumonia, since this is necessary to ensure the appropriate delivery of antibiotic therapy to each individual patient. We evaluated the results of a rapid molecular diagnostic pneumonia panel in children with LRTI in a pediatric intensive care unit (PICU). Patients and Methods: Rapid molecular diagnostic pneumonia panel (BioFire^®^, FilmArray Pneumonia Panel plus; FA-PP) findings (71 results from 46 children) in a tertiary care PICU between 2019 and 2023 were retrospectively reviewed. Results: At least one bacterial pathogen was detected in 57 cases. A total of 77% of children had underlying conditions. A total of 70.4% of children needed invasive mechanical ventilation and 54.4% had ventilator-associated pneumonia. *Pseudomonas aeruginosa* (50.8%), *Acinetobacter calcoaceticus baumannii complex* (42%), and *Klebsiella pneumoniae* (38.6%) were the most common pathogens detected with the FA-PP. Of the 33 cases diagnosed with VAP, more than one pathogen was identified in 65.9% of cases, with the most commonly identified bacteria being *K. pneumoniae* (43.1%), *P. aeruginosa* (38.6%), and *Acinetobacter calcoaceticus baumannii complex* (31.8%). According to the FA-PP results, the same antibiotic therapy was continued in 39.4% of cases, escalated in 54.5%, and de-escalated in 6.1%. Conclusions: The utilization of the FA-PP has some beneficial effects, including more prompt delivery of findings compared to conventional approaches. Additionally, this approach enables the identification of resistance profiles in children diagnosed with pneumonia in the PICU. Consequently, these test results facilitate the organization of antibiotic treatment strategies, including escalation and de-escalation approaches. The detection of resistance patterns was exclusively determined via the implementation of molecular testing, prompting a reevaluation of the isolation technique in accordance with the obtained data.

## 1. Introduction

Pneumonia is the one of the common infectious diseases and a major cause of morbidity and mortality worldwide [1]. Pneumonia is also a significant problem in hospital settings and is one of the major causes of pediatric intensive care unit (PICU) stays in children and adolescents [2,3]. Antibiotic treatment should ideally begin as soon as possible in patients diagnosed with pneumonia, since multiple studies have demonstrated a positive impact on patient survival when antibiotics are given within the first few hours [4]. A prolonged delay in the administration of antibiotics might have a negative impact on the prognosis [4]. The majority of children with pneumonia also require mechanical ventilation in the PICU [2]. Mechanical ventilation might result in decreased integrity of the alveolar–capillary membrane due to ventilator-induced lung injury, surfactant rupture and inactivation, and microvascular injury. This might affect the translocation and increased circulation of bacterial and pro-inflammatory cytokines, resulting in sepsis and/or multiple organ dysfunction [5].

Health care-associated infections are a major cause of morbidity and mortality, and 20–25% of infections are ventilator-associated pneumonia [2]. Ventilator-associated pneumonia (VAP) is defined as a nosocomial pneumonia diagnosed by the presence of new respiratory infection symptoms in patients undergoing mechanical ventilation 48 h or more after the initiation of mechanical ventilation [2]. VAP prolongs the duration of mechanical ventilation and increases costs, morbidity, and mortality [2,4]. Therefore, early diagnosis of lower respiratory tract infection and VAP, followed by prompt identification of the causative agent and appropriate treatment, is of paramount importance. Although the current therapy is often begun on an empirical basis, it is imperative that diagnostic investigations be performed to determine the microbiological etiology of the pneumonia in order to guarantee that patients receive antibiotic treatment that is successful [4].

Pneumonia care depends on a number of factors, including prompt diagnosis, appropriate therapy, and ventilation [4]. Pathogens responsible for pneumonia are notoriously difficult to detect using traditional methods, and even more so when compared to those responsible for bloodstream infections and meningitis. Hospitalized adults with pneumonia had a 38% pathogen detection rate when using conventional diagnostic procedures such standard culture, antigen detection assay, and nucleic acid detection tests [6]. Rapid syndromic molecular testing for pneumonia is now available, with assays that can detect bacteria, viruses, and antibiotic-resistance genes [7]. Testing with a molecular syndromic panel has the potential to increase analytical sensitivity, save turnaround time, and detect a wider variety of infections and resistance genes [8]. Possible benefits to patient care include less need for diagnostic tests, adjustments to antimicrobial therapy (de-escalation or cessation), and the potential for earlier removal or application of infection control precautions. There may be fewer healthcare-associated infections, shorter hospital stays, fewer unnecessary admissions and re-admissions, lower death rates, and lower overall healthcare costs as a result [8]. Molecular investigations have become the gold standard for diagnosing respiratory infections, especially viruses, in recent years due to their superior sensitivity in detecting organisms that are difficult to separate, less viable, or present in extremely minute numbers [7]. Antibiotic stewardship and the effectiveness of antimicrobial treatment could be strengthened by the data provided by molecular diagnostics on the prevalence of antibiotic-resistant genes [9]. The BioFire^®^ FilmArray Pneumonia Panel (FA-PP) which is a rapid molecular test approved by FDA, can detect a wide variety of clinically relevant targets and resistance markers in bronchoalveolar lavage specimens and sputum (including endotracheal aspirate). There is a total of 15 distinct bacteria (semiquantitative data provided), 3 atypical bacteria, 9 viruses, and 7 distinct antibiotic-resistance genes [9,10]. Although there is evidence of the efficacy of the FA-PP in the adult setting for rapidly diagnosing the complicated microbiological etiologies of pneumonia infections, there are limited data in the PICU situation [3,9,10,11,12,13,14,15,16,17]. The purpose of this research was to evaluate the performance of a rapid molecular diagnostic pneumonia panel, the FA-PP, for use with children in the PICU who have lower respiratory tract infections.

## 2. Materials and Methods

This retrospective study was conducted in the tertiary, 18-bed, pediatric intensive care unit (PICU) of Eskisehir Osmangazi University Medical Faculty between December 2019 and June 2023. This study was approved by the Local Ethical Committee of Clinical Research of Eskisehir Osmangazi University Faculty of Medicine (20/12/2022-31). All procedures performed in this trial were in accordance with the ethical standards of the institutional and/or national research committee and with the 1964 Helsinki Declaration and its later amendments or comparable ethical standards. We retrospectively evaluated medical records of hospitalized children with lower respiratory tract infection, including community-acquired pneumonia, ventilator-associated pneumonia, and pneumonia with pleural effusion or empyema, and selected children who had a rapid molecular diagnostic pneumonia panel. The clinical and demographical findings, including the presence of chronic underlying conditions, antibiotic use, the presence of central venous and urinary catheters, the presence of thoracic tubes, the need for total parenteral nutrition, the need for mechanical ventilation, and length of stay, were retrospectively reviewed.

The BioFire^®^ FilmArray Pneumonia Panel plus (FA-PP, BioMérieux) was utilized in our institution for expedited molecular diagnosis of pneumonia during the trial period. The FA-PP assay is a syndrome-specific, cartridge-based, multiplex polymerase chain reaction (PCR) method that integrates all stages of molecular diagnostics in an automated fashion, yielding results within an estimated timeframe of 1 h. The panel consisted of a total of 15 bacteria, which were reported in a semi-quantitative manner. These bacteria included *Acinetobacter calcoaceticus baumannii complex*, *Enterobacter cloacae complex*, *Escherichia coli*, *Haemophilus influenzae*, *Klebsiella aerogenes*, *Klebsiella oxytoca*, *Klebsiella pneumoniae group*, *Moraxella catarrhalis*, *Proteus* spp., *Pseudomonas aeruginosa*, *Serratia marcescens*, *Staphylococcus aureus*, *Streptococcus agalactiae*, *Streptococcus pneumoniae*, and *Streptococcus pyogenes*. Additionally, there were three atypical bacteria present in the panel: *Chlamydia pneumoniae*, *Legionella pneumophila*, and *Mycoplasma pneumoniae*. Furthermore, the panel included nine viruses—specifically, adenovirus, coronavirus, human metapneumovirus, human rhinovirus/enterovirus, influenza A, influenza B, parainfluenza virus, respiratory syncytial virus, and Middle East respiratory syndrome coronavirus. Furthermore, alongside the aforementioned bacteria and viruses, there are seven antimicrobial-resistance genes that deserve attention. These include methicillin-resistance genes (mecA/C and MREJ), carbapenemases (bla KPC, bla NDM, bla OXA-48-like, bla VIM, and bla IMP), and extended-spectrum β-lactamases (ESBL) such as bla CTX-M. The detection of antibiotic-resistance genes was reported in cases where the corresponding bacteria associated with the gene was also identified in the sample [18].

Based on clinical and laboratory data, patients who received mechanical ventilation for more than 48 h in the PICU were diagnosed with ventilator-associated pneumonia [19,20]. A new or progressive infiltrate, consolidation, cavitation, or pleural effusion on chest radiography with at least one episode of fever (>38 °C) without any other known cause, leukopenia (4000 white blood cells (WBC)/mm^3^), or leukocytosis (12,000 WBC/mm^3^) and at least two signs of new-onset purulent sputum were all necessary for VAP to be diagnosed. These warning signs included a change in the characteristics of the sputum, an increase in respiratory secretions or the need for suctioning, a new or worsening cough, tachypnea or dyspnea, rales or bronchial breath sounds, or a worsening gas-exchange profile (i.e., O_2_ desaturation (PaO_2_/FiO_2_ level 240), an increase in oxygen consumption, or requiring mechanical ventilation) [19,20]. Statistical Package for Social Sciences (SPSS) version 28.0 for Windows (SPSS, Chicago, IL, USA) was used for the statistical analysis. Continuous variables are expressed as medians (minimum–maximum). Categorical variables are shown as percentages.

## 3. Results

We evaluated the results of 93 tracheal aspirate/bronchoalveolar lavage fluid and pleural fluid samples were collected from 57 patients. No pathogens were detected by the rapid molecular diagnostic pneumonia panel in 22 of the 11 patients. Of the remaining 46 patients, at least one respiratory pathogen was detected by rapid molecular diagnostic pneumonia panel in 71 samples collected at different times. There were 63 tracheal aspirate samples from 39 patients; 33 of these patients had ventilator-associated pneumonia and 6 of them had community-acquired pneumonia. Eight pleural fluid samples were obtained from seven patients.

We analyzed medical records of 46 patients (24 boys and 22 girls) aged between 2 and 210 months. Underlying chronic comorbid conditions were present in 71% of all cases, with neurological diseases being the most common (48%), followed by cardiac (10.8%) and metabolic diseases (6.5%). Central catheters were present in 71%, urinary catheters in 32%, chest tubes in 26% and tracheostomy in 17% of cases. Mechanical ventilation was required in 74%, total parenteral nutrition in 37%, sedation in 74%, and inotropic support in 61% of cases. Pleural effusion was present in 12 cases (28%). Ventilator-associated pneumonia was diagnosed in 33 cases (70%) (Table 1).

**Figure 1:**
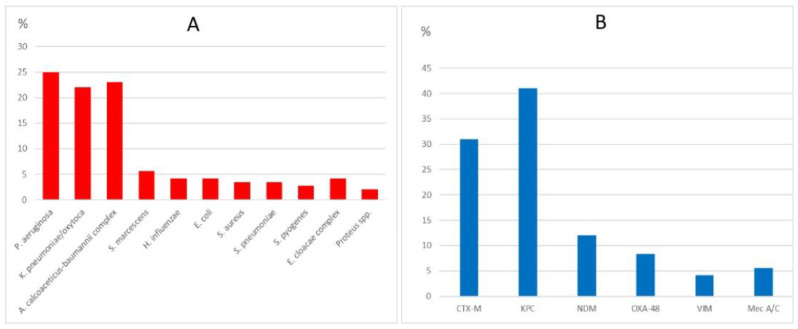
The proportional distribution of the detected bacteria (**A**) and antibiotic-resistance genes (**B**). CTX-M: cefotaximase-Munich, KPC: Klebsiella pneumoniae carbapenemase, NDM: New Delhi metallo-beta-lactamase, OXA-48: OXA-48 carbapenemase, VIM: Verona integron-encoded metallo-beta-lactamase, Mec-A/C: methicillin-resistance gene.

Of the 71 samples tested using the FA-PP, 35 (49%) were positive for a single respiratory pathogen and 36 (51%) were positive for multiple respiratory pathogens. A total of 142 respiratory pathogens were detected. The most common bacterial pathogens were *Pseudomonas aeruginosa* (25%), *Klebsiella pneumoniae* (22%), *Acinetobacter baumannii complex* (23%), and *Serratia marcescens* (5.6%). The most common viral pathogens were rhinovirus/enterovirus (40%) and respiratory syncytial virus (RSV) (14%). The most commonly detected antibiotic-resistance genes were KPC (41%), CTX-M (31%), NDM (12%), and OXA-48 (8.4%) (Table 2). The proportional distribution of the detected bacteria and antibiotic-resistance genes is shown in Figure 1.

According to the FA-PP results, the same antibiotic therapy was continued in 35% of cases, escalated in 56%, and de-escalated in 8.4%.

Of the 33 cases diagnosed with ventilator-associated pneumonia, 28 (84%) had an underlying chronic comorbid condition, with neurological disease being the most common (42%), followed by cardiac disease (15%). Tracheostomy was present in 72%, a chest tube in 15%, and pleural effusion in 15% of these cases. The 28 day mortality rate was 21.2%. In cases diagnosed with ventilator-associated pneumonia, multiple pathogens were detected in 65.9% of cases, with the most commonly identified pathogens being *Klebsiella pneumoniae* (43.1%), *Pseudomonas aeruginosa* (38.6%), and *Acinetobacter calcoaceticus-baumannii complex* (31.8%). The most common viral pathogen was rhinovirus/enterovirus (20.8%). The detected antibiotic-resistance genes were KPC (38.6.5%), CTX-M (25.0%), NDM (6.8%), OXA-48 (6.8%), and Mec-A/C (6.8%) (Table 3). According to the FA-PP results, the same antibiotic therapy was continued in 39.4% of cases, escalated in 54.5%, and de-escalated in 6.1%.

## 4. Discussion

The rapid molecular diagnostic pneumonia panel, FilmArray Pneumonia Panel plus (FA-PP), was able to identify at least one respiratory infection in 71 of the 46 patients’ samples. Out of the 33 patients diagnosed with VAP, more than half (65.9%) had multiple pathogens detected; *K. pneumonia P. aeruginosa*, and *Acinetobacter calcoaceticus-baumannii complex* were the most frequently isolated pathogens. There is only one prospective observational study about the evaluation of the FA-PP in children with VAP in the PICU [17]. They detected 46 potentially pathogenic bacteria in bronchial samples of intubated children in a PICU in Morocco from March to November 2021, with a sensitivity of 93%, specificity of 90%, negative predictive value of 100%, and positive predictive value of 23%. The most frequent bacterium was *Moraxella catarrhalis* (11.4%) and the most frequent virus was rhinovirus/enterovirus. Regarding the FA-PP results, they changed in antibiotic therapy in 39.5% of the patients. In a study conducted by Kamel et al. [3], it was demonstrated that the predominant pathogens identified through the FA-PP were *K. pneumoniae* (28 out of 50 samples), followed by *Acinetobacter baumannii complex* (18 out of 50 samples) and *P. aeruginosa* (12 out of 50 samples) among adult patients, and these findings align with our own study.

The FA-PP has been authorized by the Food and Drug Administration (FDA) for the purpose of detecting and identifying various respiratory viruses and bacterial pathogens in sputum or bronchial alveolar lavage (BAL)-like specimens obtained from individuals exhibiting symptoms indicative of lower respiratory tract infections. The test in question aims to target a total of 18 distinct bacteria, 9 distinct viruses, and 7 distinct antibiotic-resistance genes [11]. In order to evaluate the effectiveness of the FA-PP in detecting infections in lower respiratory tract specimens obtained from adult patients in the emergency department and intensive care unit, Webber et al. [11] conducted a study to test its diagnostic yield and accuracy. The FA-PP method demonstrated a 58.5% accuracy in properly identifying viral or bacterial targets in the samples. They observed that a significant majority of samples (48.5%) tested positive for a bacterial pathogen; additionally, 14% of the samples exhibited viral targets, with 4% of specimens showing codetection of both bacterial and viral pathogens. The findings of this adult cohort exhibited similarities to our investigation, despite there being notable differences in terms of patient characteristics and study design. In a study conducted by Kamel et al. [3], the authors examined the use of mini-bronchoalveolar specimens in the evaluation of the FA-PP in 50 adult patients diagnosed with hospital-acquired pneumonia. The study was conducted in an intensive care unit located in a tertiary care hospital in Egypt. When compared to conventional culture methods, the FA-PP demonstrated a sensitivity and specificity of 100% and 90%, respectively, and the FA-PP semi-quantitative analysis demonstrated a concordance rate of 47.4% among positive-culture specimens. Ginocchio et al. [10] observed comparable findings, with a positive predictive accuracy of 93% and a negative predictive accuracy of 96%. Lee et al. [9] conducted an evaluation of the efficacy of the FA-PP in identifying respiratory infections and determining resistance factors. The researchers collected a total of 59 endotracheal aspirates and bronchoalveolar lavage specimens from 51 adult patients who were admitted to intensive care units. The FA-PP method demonstrated efficacy in the identification of respiratory bacterial infections, as evidenced by an overall positive agreement of 90% (with a 95% confidence interval of 73.5% to 97.9%) and a negative agreement of 97.4% (with a 95% confidence interval of 96.0% to 98.4%). The FA-PP revealed a concordance rate of 53.6% for the specimens that tested positive in culture and 86.3% for the specimens that tested negative in culture. A potential limitation of the FA-PP assay is the absence of fungal targets, as well as some significant pathogens in the PICU like *Stenotrophomonas maltophilia* [11]. In our study, we checked our negative test for the FA-PP but did not detect *S. maltophilia* among these children. A possible issue arises from the absence of *S. maltophilia* in the FA-PP, as this bacterium is frequently seen among children in Türkiye and is known for its multidrug-resistant properties [21].

Rapid molecular diagnostic pneumonia panels can detect bacteria far sooner than traditional culture methods, allowing for more precise control over when and how many antibiotics are administered (through escalation and de-escalation) [7,8]. Webber et al. [11] showed a notable advantage over culture-based approaches by enabling the identification of bacterial pathogens and resistance indicators in 44.2 h and 56.3 h less time, respectively, through the utilization of real-time specimen analysis. Empiric antibiotic selection during intensive care unit stays varies according to the patient’s previous infection and the infectious agent. Recent infection with MDR organisms within 90 days changes the empiric antibiotic treatment strategies [20]. Appropriate antibiotic therapy is essential in any setting, and preventing the spread of antibiotic-resistant bacteria is a global priority. Webber et al. [11] demonstrated 100% PPA between the FA-PP and the standard of care in the detection of antimicrobial resistance, suggesting high assay sensitivity. According to the findings of Webber et al. [11], six of the samples tested exhibited positivity only for blaCTX-M, whereas one sample showed positivity for both blaCTX-M and blaKPC. Additionally, another sample was found to be positive exclusively for blaKPC, as determined using FA-PP analysis. In the study conducted by Lee et al. [9], the assessment of antibiotic-resistance genes was performed using the FA-PP. The results revealed a PPA of 97%, with a negative NPA agreement of 95% compared to normal antibiotic sensitivity testing. Debbagh et al. [17] demonstrated that the identification of resistance genes revealed a significant prevalence of carbapenemases (65.2%) and ESBLs (34.8%) using the FA-PP in a pediatric setting. This observation can be attributed to the administration of previous, frequently broad-spectrum, antibiotic treatment among critically ill patients admitted to the intensive care unit. In our study, KPC and CTX-M were the genes most often found for antibiotic resistance, followed by NDM, OXA-48, and mecA/C, and the majority of the enrolled patients had an underling condition and risk factors related to the intensive care unit. These molecular tests are the only ones capable of detecting resistance patterns at the genetic level in our setting, and we regularly reviewed isolation protocols for each patient in light of the findings. According to Lee et al. [11], the utilization of the FA-PP as a rapid prediction tool facilitated the infection control personnel in identifying the patients who required cohort placement and isolation from the general ward population. Regarding the detection of antimicrobial-resistance genes, Kamel et al. [3] demonstrated that the FA-PP exhibited a considerable level of concordance in comparison to VITEK-2 breakpoints. Their study and our study showed a greater rate of detection for carbapenemase production and ESBLs. It is possible that the widespread usage of antimicrobial medicines among intensive care unit patients is to blame for this increased resistance rate; this increased selective pressure has allowed for the emergence of new resistant phenotypes [9,11]. This presents a promising prospect for enhancing the management, prevention, and surveillance of antibiotic resistance.

Although empirical antibiotics are essential for individuals with pneumonia, their efficacy is at risk due to their widespread usage [11]. The potential of rapid diagnostic testing for pneumonia lies in its ability to inform therapeutic decisions and decrease the utilization of broad-spectrum antibiotics. However, the effectiveness of these test methods is contingent upon their accuracy. The utilization of the FA-PP facilitates the differentiation between the commensal and pathogenic states of bacteria by enabling the identification of these microorganisms at semi-quantitative thresholds. According to Debbagh et al. [17], there is a positive correlation between the threshold level and the likelihood of a bacterium being associated with a lower respiratory tract infection. Sandulescu et al. [22] proposed the implementation of a diagnostic and antimicrobial stewardship approach specifically targeting patient populations deemed to be at a heightened risk of acquiring multidrug-resistant bacterial infections. The authors emphasize the importance of selecting appropriate diagnostic tests, utilizing suitable sample types, and ensuring timely collection of samples, all of which are crucial to informing treatment decisions [22]. In our study, according to the rapid molecular diagnostic pneumonia panel results, the same antibiotic therapy was continued in 35% of cases, escalated in 56%, and de-escalated in 8.4%, and isolation procedures were implemented for children with resistance patterns. According to Lee et al. [11], the findings of the FA-PP have the potential to influence antibiotic prescription decisions in 40.7% of patients. The authors indicated that the findings of the FA-PP may have resulted in a reduction in initial empirical antibiotic treatment in 16 patients, accounting for 27.1% of the total sample. Additionally, in nine patients (13.6%), there was a need to escalate or introduce an additional effective antibiotic. However, in the majority of cases (55.9%), no modifications to the antibiotic regimen were deemed necessary.

In this study period, we utilized the FA-PP panel on a series of seven consecutive cases with pleural effusion, with or without empyema. It is worth noting that the manufacturer’s guidelines do not endorse the application of this test on pleural fluid. Invasive GAS infection is supported by other clinical symptoms and traditional culture results of these individuals, and we were able to obtain positive results for *Streptococcus pyogenes* in four children [23]. *Haemophilus influenzae* was detected in a previously asymptomatic male patient presenting with empyema, as confirmed by pleural fluid analysis utilizing the FA-PP method. Positive results were obtained from conventional culture of both pleural fluid and blood samples. Furthermore, molecular testing classified these samples as “non-typeable.” Two individuals were found to have tested positive for Streptococcus pneumoniae, with one of them also exhibiting a positive result in the conventional pleural fluid culture. Additional comprehensive investigations are warranted to enhance our understanding of the potential utility of the FA-PP in the evaluation of pleural fluid.

We had some limitations due to the retrospective single-center study design. We did not perform a concomitant conventional respiratory sample culture for all patients. Multiplex PCR is a new molecular biology technique that is supposed to have better sensitivity than conventional culture. We evaluated the performance of the FA-PP among patients with suspected lower respiratory infection in the PICU; however, our setting was a tertiary care center and the majority of the patients had underlying conditions and risk factors for serious infection. The infections of the patients enrolled in the study were highly heterogeneous, including community-acquired pneumonia, health care-associated pneumonia, and ventilator-associated pneumonia.

## 5. Conclusions

The BioFire^®^ FilmArray Pneumonia Plus panel has the potential to offer timely insights into causative microorganisms. The quick molecular diagnostic pneumonia panel offers several advantages in the context of pediatric patients with LRTI or VAP in the PICU. The potential application of semi-quantitative detection methods for 15 bacterial targets and their associated antibiotic-resistant genes could potentially enhance the implementation of antimicrobial stewardship programs and improve infection control measures in the PICU. These advantages include the ability to provide faster results compared to conventional diagnostic approaches, as well as the capability to detect the resistance profile of pathogens causing pneumonia in this specific population. One of the main disadvantages of this panel is that it is high cost and difficult to implement in every center. There is a need for multicenter, prospective, and cost-effective studies involving pediatric intensive care patients.

## Figures and Tables

**Table 1:** Clinical characteristics of 46 children with positive results with the FilmArray Pneumonia Plus panel.

Patients	(n = 46)
**Age (month)**	71 (2–210)
**Comorbidities (n/%)**	33 (71.7%)
Neurological disease	22 (47.8%)
Cardiac disease	5 (10.8%)
Metabolic disease	3 (6.5%)
Hemato-oncological disease	2 (4.3%)
Rheumatological disease	1 (2.1%)
**Central catheter n (%)**	33 (71%)
**Urinary catheter n (%)**	15 (32%)
**Mechanical ventilation n (%)**	34 (74%)
**Total parenteral nutrition n (%)**	17 (37%)
**Tracheostomy n (%)**	8 (17%)
**Sedo-analgesia n (%)**	34 (74%)
**Inotrope n (%)**	28 (61%)
**Presence of thorax tube n (%)**	12 (26%)
**Pleural effusion n (%)**	13 (28%)
**Ventilator-associated pneumonia n (%)**	33 (70%)
**Mortality (first 28 days)**	13 (28%)

**Table 2:** Distribution of pathogens detected by the FilmArray Pneumonia panel plus.

Rapid Molecular Diagnostic Pneumonia Panel Test	Total (n = 71)
**Single pathogen/multiple pathogens**	35/36 (49% vs. 51%)
**Total microorganisms**	142
**Bacteria**	
*Pseudomonas aeruginosa*	35 (25%)
*Klebsiella pneumoniae-oxytoca*	31 (22%)
*Acinetobacter calcoaceticus-baumannii complex*	33 (23%)
*Serratia marcescens*	8 (5.6%)
*Haemophilus influenzae*	6 (4.2%)
*Escherichia coli*	6 (4.2%)
*Staphylococcus aureus*	5 (3.5%)
*Streptococcus pneumoniae*	5 (3.5%)
*Streptococcus pyogenes*	4 (2.8%)
*Enterobacter cloacae complex*	6 (4.2%)
*Proteus* spp.	3 (2.1%)
**Antibiotic resistance**	41/71
One/multiple	21/20
CTX-M (cefotaximase-Munich)	22 (31%)
KPC (Klebsiella pneumoniae carbapenemase)	29 (41%)
NDM (New Delhi metallo-beta-lactamase)	9 (12%)
OXA-48 (OXA-48 carbapenemase)	6 (8.4%)
VIM (Verona integron-encoded metallo-beta-lactamase)	3 (4.2)
Mec-A/C (methicillin-resistance gene)	4 (5.6%)
**Viruses**	n:35
One/multiple	25/3
Rhinovirus/enterovirus	14 (40%)
RSV	5 (14%)
Human metapneumovirus	4 (11%)
Adenovirus	8 (22%)
Influenza A/B	4 (11%)

**Table 3:** Clinical characteristics, distribution of pathogens, and percentage of antibiotic resistance of 33 children with ventilator-associated pneumonia and positive results with the FilmArray Pneumonia Panel plus.

Patients with VAP	Total (n = 33)
**Age (median, minimum–maximum)**	66 (2–210)
**Gender (boy/girl)**	
Comorbidities	28 (84%)
Neurological disease	14 (42%)
Cardiac disease	5 (15%)
Metabolic disease	4 (12%)
Hemato-oncological disease	2 (6%)
**Central catheter n (%)**	29 (88%)
**Total parenteral nutrition n (%)**	16 (49%)
**Enteral nutrition n (%)**	32 (97%)
**Sedo-analgesia n (%)**	31 (94%)
**Inotrope n (%)**	19 (58%)
**Tracheostomy n (%)**	9 (27%)
**ANTIBIOTIC TREATMENT**	
Continuation (same as)	13 (39.4%)
Escalation	18 (54.5%)
De-escalation	2 (6.1%)
POSITIVE FA-PP RESULTS	N = 44
Single/multiple pathogens	15/29
**Bacteria**	
*Pseudomonas aeruginosa*	17 (38.6%)
*Klebsiella pneumoniae-oxytoca*	19 (43.1%)
*Acinetobacter calcoaceticus-baumannii complex*	14 (31.8%)
*Serratia marcescens*	7 (15.9%)
*Haemophilus influenzae*	2 (4.5%)
*Escherichia coli*	5 (11.3%)
*Staphylococcus aureus*	4 (9.0%)
*Streptococcus pneumoniae*	2 (4.5%)
*Enterobacter cloacae complex*	3 (6.8%)
*Proteus* spp.	1 (2.2%)
**Antibiotic resistance**	
CTX-M (Cefotaximase-Munich)	11 (25.0%)
KPC (Klebsiella pneumoniae carbapenemase)	17 (38.6%)
NDM (New Delhi metallo-beta-lactamase)	3 (6.8%)
OXA-48 (OXA-48 carbapenemase)	3 (6.8%)
VIM (Verona Integron-encoded Metallo-beta-lactamase)	0 (%)
Mec-A/C (Methicillin resistance gene)	3 (6.8%)
**Viruses**	16 (25%)
One/multiple	13/3
Rhinovirus/enterovirus	9 (20.4%)
RSV	3 (6.8%)
Human metapneumovirus	2 (4.5%)
Adenovirus	4 (9.0%)
Influenza A/B	3 (6.8%)

## Data Availability

The data are available from the corresponding author upon reasonable request.
